# TREM2 Acts as a Tumor Suppressor in Colorectal Carcinoma through Wnt1/*β*-catenin and Erk Signaling

**DOI:** 10.3390/cancers11091315

**Published:** 2019-09-06

**Authors:** Su-Man Kim, Eun-Mi Kim, Kon-Young Ji, Hwa-Youn Lee, Su-Min Yee, Su-Min Woo, Ja-Woon Yi, Chul-Ho Yun, Harim Choi, Hyung-Sik Kang

**Affiliations:** 1 School of Biological Sciences and Technology, Chonnam National University, Gwangju 500-757, Korea (S.-M.K.) (S.-M.Y.) (S.-M.W.) (J.-W.Y.) (C.-H.Y.); 2 Korea Institute of Toxicology, Daejeon, 34114, Korea; 3 Herbal Medicine Research Division, Korea Institute of Oriental Medicine, Daejeon 34054, Korea; 4 Medical Device Development Center, Daegu-Gyeongbuk Medical Innovation Foundation, Daegu 701-310, Korea; 5 Department of Nursing, Nambu University, Gwangju 506-706, Korea

**Keywords:** TREM2, colorectal cancer, Wnt/*β*, *β* catenin, Erk, tumor suppression

## Abstract

TREM2 (triggering receptor expressed on myeloid cells) is involved in the development of malignancies. However, the function of TREM2 in colorectal cancer has not been clearly elucidated. Here, we investigated TREM2 function for the first time in colorectal epithelial cancer cells and demonstrated that TREM2 is a novel tumor suppressor in colorectal carcinoma. Blockade of TREM2 significantly promoted the proliferation of HT29 colorectal carcinoma cells by regulating cell cycle-related factors, such as p53 phosphorylation and p21 and cyclin D1 protein levels. HT29 cell migration was also increased by TREM2 inhibition via MMP9 (matrix metalloproteinase 9) expression upregulation. Furthermore, we found that the tumor suppressor effects of TREM2 were associated with Wnt/*β*-catenin and extracellular signal-regulated kinase (ERK) signaling. Importantly, the effect of TREM2 in the suppression of tumor development was demonstrated by in vivo and in vitro assays, as well as in human colon cancer patient tissue arrays. Overall, our results identify TREM2 as a potential prognostic biomarker and therapeutic target for colorectal cancer.

## 1. Introduction

TREMs are type I membrane proteins with an extracellular Ig-like domain and a short cytoplasmic tail that have no intrinsic signaling capacity [1]. TREM signaling relies on association with the DNAX-activating protein 12 (DAP12) cytosolic adapter [2]. DAP12 contains an immunoreceptor tyrosine-based activation motif (ITAM) [1], which becomes phosphorylated upon the activation of DAP12-associated receptors. Phosphorylated ITAM in turn recruits and activates spleen tyrosine kinase (Syk), leading to cellular responses such as the regulation of cytokine production [3,4]. TREM family members play distinct roles in regulating inflammatory responses [1]. For example, the activation of TREM1 amplifies inflammation [5]. In contrast, TREM2 signaling suppresses the inflammatory reaction [1,6,7]. The role of TREM3 in innate immunity has not been elucidated.

TREM2 is a cell surface receptor and a member of the Ig superfamily with one V-type extracellular domain, a charged transmembrane domain, and a short cytoplasmic tail [8]. TREM2 is expressed in monocytes [9], macrophages [10,11], microglia [8,12], dendritic cells (DCs) [13], and osteoclasts [14]. Furthermore, TREM2/DAP12 signaling induces the phosphorylation of extracellular signal-regulated kinase (ERK) and promotes the upregulation of CC chemokine receptor 7 (CCR7) in dendritic cells [13]. In addition, the inhibition of PI3 kinase suppresses TREM2 expression [15]. Although these results suggest that the TREM2/PI3 kinase signaling pathway negatively regulates TREM-2 expression, homeostasis maintenance, TREM2 ligand(s) and TREM2 functions in apoptosis are not clearly understood.

More than 90% of all colon cancers will have an activating mutation of the canonical Wnt signaling pathway, and the dysregulation of the Wnt/*β*-catenin pathway plays an essential role in colon carcinogenesis [16]. Activated GSK (glycogen synthase kinase) -3*β* phosphorylates target proteins such as *β*-catenin, which is a transcription factor that regulates the expression of several genes, including c-myc and cyclin D1, by translocation into the nucleus, where it interacts with transcription factors of the TCF/LEF-1 (T-cell factor/lymphoid enhancer-binding factor family-1) [17,18]. Interestingly, both ERK and Wnt signaling pathways are involved in the regulation of differentiation, organogenesis, adipogenesis, and proliferation, and the aberrant regulation of the Wnt/*β*-catenin signaling pathway, including the upregulation of *β*-catenin, is one of the major causes of colon and other cancers [19]. PI3K-activated AKT (protein kinase B) can phosphorylate GSK-3*β*, leading to the inactivation of GSK-3*β* and augmentation of *β*-catenin-TCF4 (T-cell factor 4) transcriptional activity [20]. Recent studies have indicated that the crosstalk between the PI3K/AKT pathway and the GSK-3*β*/*β*-catenin pathway and between the ERK pathway and the GSK-3*β*/*β*-catenin pathway plays a role in cell survival and proliferation [19,21,22], and TREM2 is one of the molecules that may regulate GSK-3*β*/*β*-catenin signaling through these pathways. However, the underlying mechanisms by which TREM2 regulates Wnt signaling in colon cancer cells are completely unknown.

In this study, we demonstrated that TREM2 inhibits the proliferation of colon cancer cells by attenuating cell cycle progression by downregulating cyclin D1 expression. Furthermore, TREM2 increases *β*-catenin phosphorylation by activating GSK-3*β*, which suppresses *β*-catenin accumulation and TCF4 transcriptional activity, resulting in the downregulation of tumorigenicity in colon cancer cells.

## 2. Results

### 2.1. TREM2 Suppresses the Proliferation of HT29 Colon Cancer Cells by Downregulating Cell Cycle Progression

**Figure 1:**
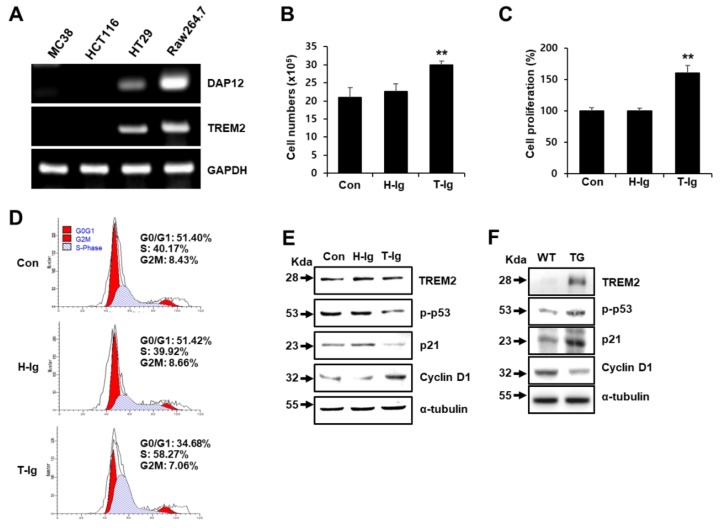
Blockade of triggering receptor expressed on myeloid cells (TREM)2 promotes the growth of HT29 colon cancer cells. (**A**) Using RT-PCR, mRNA expression levels of DNAX adaptor protein-12 (DAP12) and TREM2 were measured in MC38, HCT116, and HT29 colon cancer cell lines, and the Raw264.7 macrophage cell line was used as a positive control. (**B**) HT29 cells (1 × 10^6^ cells) were plated in each well of six-well plates and treated with H-Ig or T-Ig (2 μg/mL) for 48 h. Cell number was assessed by the trypan blue dye exclusion method, (**C**) and cell proliferation was analyzed by 3-(4,5-dimethylthiazol-2-yl)-5-(3-carboxymethoxyphenyl)-2-(4-sulphophenyl)-2H-tetrazolium) (MTS) assay. (**D**) Cell cycle analysis in HT29 cells treated with hIg or TREM2-Ig (2 μg/mL) was performed by flow cytometry. (**E**) Cell cycle-related protein expression in HT29 cells (**F**) and in cells isolated from the colons of WT littermate and TREM2-TG mice was analyzed by Western blot. The data are representative of three independent experiments (** *p* < 0.01).

Previous studies have reported that TREM2 expression is observed in non-myeloid cell types, including hepatic endothelial cells and epithelial cells of the genitourinary tract [15,23]. However, the expression and function of TREM2 have not been investigated directly in colorectal epithelial cells. Here, we first confirmed the expression of TREM2 in epithelial colorectal carcinoma cell lines, such as MC38, HCT116 and HT29, and compared it to that in the Raw 264.7 myeloid cell line. TREM2 and DAP12 expression was clearly observed in HT29 and Raw 264.7 cells but not in MC38 and HCT116 cells (Figure 1A). To examine the effect of TREM2 on the proliferation of HT29 cells, trypan blue dye exclusion and MTS (3-(4,5-dimethylthiazol-2-yl)-5-(3-carboxymethoxyphenyl)-2-(4-sulphophenyl)-2H-tetrazolium) assays were performed. The growth of HT29 cells was increased by treatment with TREM2-Ig (T-Ig), a neutralizing agent, compared with that of control cells (Figure 1B). In addition, the cell proliferation rate according to an MTS assay was induced by treatment with T-Ig (Figure 1C). Given the close relationship of cell proliferation with cell cycle progression, alterations in cell cycle distribution were investigated. Treatment with T-Ig remarkably induced the S phase (40% to 58%) and decreased the G0/G1 (51% to 34%) and G2/M phase (8% to 7%) (Figure 1D). Next, to determine whether the induction of HT29 cell proliferation by blocking TREM2 was directly related to the altered expression of cell cycle regulatory proteins, the expression of p53, p21 and cyclin D1 was analyzed by Western blot. Compared with control cells, HT29 cells treated with T-Ig showed decreased p53 phosphorylation (p-p53) and p21 protein levels but increased cyclin D1 expression (Figure 1E). In addition, increased p-p53 and p21 expression and decreased cyclin D1 expression were shown in colon cells from TREM2 transgenic (TREM2-TG) mice compared with the expression in wild-type (WT) mice (Figure 1F). These data suggest that TREM2 suppresses the proliferation of HT29 cells by inducing cell cycle arrest through the regulation of cell cycle regulators.

### 2.2. TREM2 Suppresses the Metastatic Potential of HT29 Colon Cancer Cells

**Figure 2:**
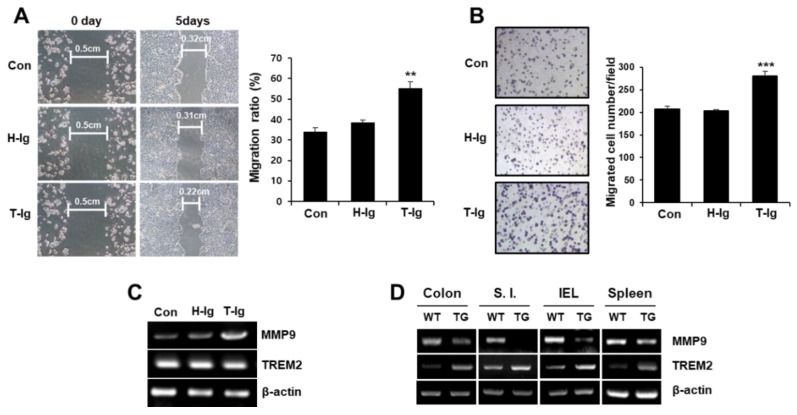
Inhibition of TREM2 enhances the migration of HT29 cells. (**A**) HT29 cells (1 × 10^5^ cells) treated with h-Ig or T-Ig (2 μg/mL) were plated in each well of six-well plates and allowed to grow to confluence for 24 h. Confluent monolayers were scratched with a pipette tip and cultured for 5 days. The scratch wound areas were quantitated from the photographs using a computer image analysis system. The migration ratio was calculated and represented as bar graphs. (**B**) Matrigel invasion assays were performed as described in the Materials and Methods, and the results are expressed as a bar graph representing the mean number of invaded cells. (**C**) Expression of MMP9 in HT29 cells (**D**) and cells isolated from various tissues of WT littermate and TREM2-TG mice was analyzed by RT-PCR. The data are representative of three independent experiments (** *p* < 0.01, *** *p* < 0.001).

To explore the effect of TREM2 on the metastatic potential of colon cancer cells, we performed scratch wound healing and Matrigel invasion assays. We found that the wound healing ability was enhanced by treatment with T-Ig compared with that under control conditions (Figure 2A). Furthermore, the number of invaded cells was significantly higher in the presence of T-Ig than in control conditions (Figure 2B). In addition, the expression of MMP9, an important metalloproteinase in the invasion and metastasis of cancer cells, was dramatically upregulated by treatment with T-Ig (Figure 2C). The expression of MMP9 was also downregulated in various organs of TREM2-TG mice (Figure 2D). These data suggest that TREM2 suppresses the metastatic potential of HT29 cells by downregulating MMP9 expression.

### 2.3. TREM2 Regulates the Expression of Wnt/β-Catenin Signaling-Related Molecules

**Figure 3:**
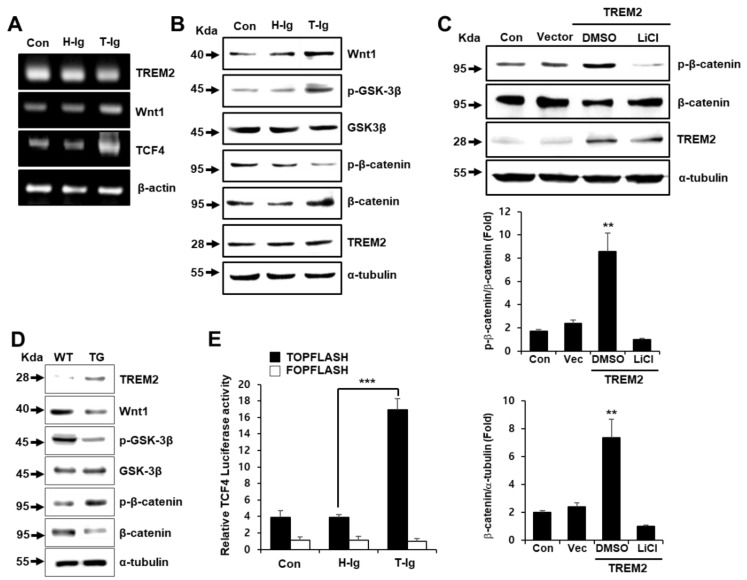
TREM2 suppresses the Wnt/*β*-catenin signaling pathway in HT29 cells. (**A**) The indicated mRNA expression levels were analyzed by RT-PCR, (**B**) and the expression levels of Wnt/*β*-catenin signaling-related proteins were measured by Western blot in HT29 cells treated with H-Ig or T-Ig (2 μg/mL) for 24 h. (**C**) Western blot analysis was performed using protein from HT29 cells transfected with plasmids containing the TREM2 gene or vector. The GSK-3*β* inhibitor LiCl (1 μM) was added for 10 min before analysis. The relative protein expression was quantitatively analyzed by densitometry (lower panel). (**D**) The expression levels of the indicated proteins in cells isolated from the large intestine of WT littermate and TREM2-TG mice were determined by Western blot. (**E**) TCF4 luciferase activity was determined in HT29 cells treated with H-Ig or T-Ig (2 μg/mL) for 24 h. The data are representative of three independent experiments (** *p* < 0.01, *** *p* < 0.001).

The aberrant activation of the Wnt/*β*-catenin pathway is involved in the pathogenesis of colon cancer [24]. Therefore, the Wnt/*β*-catenin signaling pathway is regarded as a target for anti-cancer therapeutic agents. To investigate whether TREM2 can regulate the expression of Wnt and its signaling molecules, including GSK-*β*, *β*-catenin and T cell factor 4 (TCF4), we examined their expression in HT29 cells and colon cells from TREM2-TG mice. The gene expression of Wnt1 and TCF4 was upregulated in T-Ig-treated HT29 cells compared with that in control cells (Figure 3A). Furthermore, T-Ig-induced Wnt1 expression led to the increased phosphorylation of GSK-3*β* at Ser9 and suppressed the phosphorylation of *β*-catenin at Ser33 compared with control conditions (Figure 3B). In addition, the ectopic expression of TREM2 in HT29 cells resulted in a decrease in the total *β*-catenin protein level upon increasing p-*β*-catenin (Figure 3C). Importantly, treatment with LiCl, an inhibitor of GSK-*3β*, completely suppressed *β*-catenin phosphorylation and markedly increased the *β*-catenin protein level (Figure 3C). We also found that colon cells from TREM2-TG mice had decreased levels of Wnt1 protein, GSK-3*β* phosphorylation at Ser9 and *β*-catenin and increased levels of total *β*-catenin expression (Figure 3D), suggesting that TREM2 plays an important role in the regulation of the Wnt1/*β*-catenin signaling pathway. Therefore, we hypothesized that TREM2 might suppress *β*-catenin/TCF4 transactivation activity. To explore this hypothesis, we conducted a *β*-catenin/TCF4 luciferase reporter assay using reporter plasmids containing multiple binding sites (TOPflash) or mutated binding sites (FOPflash). We found that T-Ig suppression of TREM2 signaling upregulated *β*-catenin/TCF4-mediated transactivation in HT29 cells (Figure 3E).

### 2.4. TREM2 Inhibits the ERK Signaling Pathway

**Figure 4:**
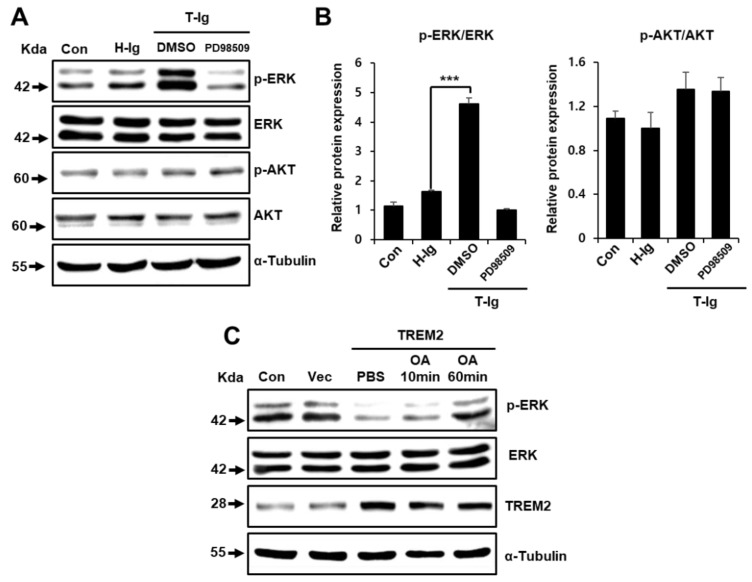
TREM2 downregulates the extracellular signal-regulated kinase (ERK) signaling pathway. (**A**) The indicated protein expression levels in HT29 cells treated with H-Ig or T-Ig (2 μg/mL) or PD98509 (10 μM) for 10 min were analyzed by Western blot. (**B**) The relative protein expression was quantitatively analyzed by densitometry. (**C**) HT29 cells were transfected with plasmids containing the TREM2 gene or vector and treated with OA (10 nM) for 10 min or 1 h, and phosphorylation levels of ERK were analyzed by Western blot. The data are representative of three independent experiments (*** *p* < 0.001).

Extracellular signal-regulated kinase (ERK) associates with and phosphorylates GSK-3*β*, which results in the inactivation of GSK-3*β* and upregulation of *β*-catenin [20]. We observed the upregulation of ERK1/2 phosphorylation upon treatment with T-Ig, which was suppressed by treatment with the MEK inhibitor PD98059 (Figure 4A). However, AKT phosphorylation was not affected by T-Ig treatment (Figure 4B). These findings agree with a previous report that the ERK-dependent or AKT-independent phosphorylation of GSK-3 is involved in the development of human colon cancers [25]. Furthermore, treatment with okadaic acid (OA), an inhibitor of protein phosphatases 1 and 2A (PP1 and 2A), increased ERK1/2 phosphorylation, which was downregulated by TREM2 overexpression (Figure 4C). These results suggest that TREM2-induced dephosphorylation of ERK might be specifically mediated by PP1 and 2A, resulting in GSK-3*β* dephosphorylation.

### 2.5. TREM2 Inhibits the Tumorigenicity of Colon Cancer Cells

**Figure 5:**
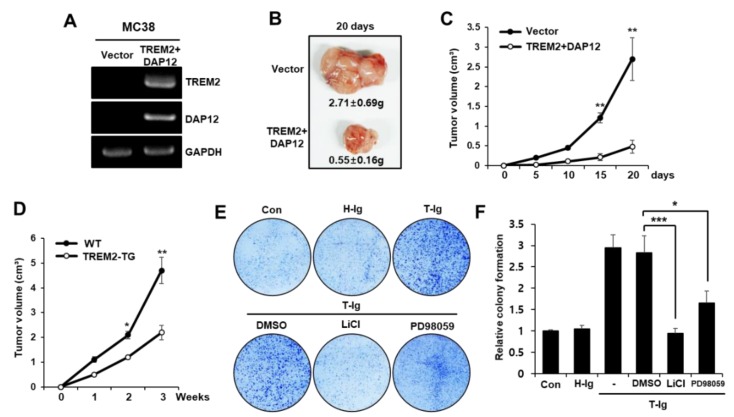
Inducing TREM2 expression suppresses tumorigenicity. (**A**) MC38 cells were transfected with plasmids containing the TREM2 gene or vector, (**B**) and the transfected MC38 cells (5 × 10^5^) were injected subcutaneously into C57BL/6J mice. Tumors were isolated from the mice after 20 days, and representative tumors are shown. (**C**) During the observation period of 20 days, the tumor volumes were measured by bilateral Vernier calipers every 5 days. (**D**) WT littermate or TREM2-TG mice were injected subcutaneously with 5 × 10^5^ MC38 cells. The tumor volumes were measured and are represented in graphs. (**E**) Colony forming assays were performed in HT29 cells treated with H-Ig or T-Ig (2 μg/mL) or LiCl (1 μM) or PD98509 (10 μM) for 24 h. (**F**) The colony formation density was measured by ImageJ, and the relative colony formation is represented as bar graphs. The data are representative of three independent experiments (** *p* < 0.01, *** *p* < 0.001).

To clarify the anti-tumorigenic effect of TREM2 in vivo, we investigated tumor incidence in WT mice injected subcutaneously with MC38 cells transfected with vector or TREM2 plasmid and DAP12 cells (Figure 5A). The injection of MC38-TREM2+DAP12 cells resulted in an approximately five-fold reduction in tumor volume compared with the injection of MC38-vector cells, implying that TREM2 overexpression dramatically suppressed tumor incidence (Figure 5B,C). Immune cell infiltration and cytokine expression critically determine the progression and development of colorectal cancer [26,27]. Therefore, we determined whether the TREM2-induced suppression of tumorigenicity might be associated with immune cells and cytokines. The distribution of tumor-infiltrated immune cells, such as CD4, CD8 T cells, NK (natural killer) cells, NK T cells, macrophages, granulocytes and dendritic cells, was similar between tumors generated by MC38-vector and MC38-TREM2+DAP12 cells (Figure S1A). Interestingly, a reduced mRNA expression of pro-tumor cytokines (IL-4 and IL-6) and bivalent property cytokines (IL-1b and IL-21) and an increased mRNA expression of anti-tumor cytokines (IL-12 and IL-15) were observed in tumors from MC38-TREM2+DAP12 cells compared with the expression in tumors from MC38-vector cells (Figure S1B). These findings clearly indicate that inducing the expression of TREM2 in colorectal cancer cells can inhibit tumorigenicity. In addition, the tumor volumes were remarkably decreased in TREM2-TG mice injected with MC38 cells compared with those in WT mice (Figure 5D). Furthermore, the tumor colonization of HT29 cells was promoted by treatment with T-Ig, and this effect was inhibited by treatment with LiCl and PD98059 (Figure 5E,F).

### 2.6. TREM2 Expression is Downregulated in Highly Proliferative Human Colon Cancers

**Figure 6:**
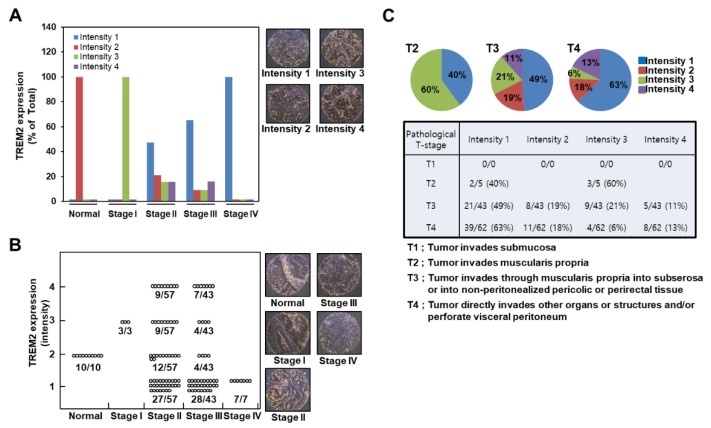
Downregulation of TREM2 expression in highly proliferative human colon carcinoma. (**A**) The expression levels of TREM2 in a human colon carcinoma tissue array were visualized by immunohistochemical analysis, (**B**) and the intensity was classified as grades of 1, 2, 3 and 4. The intensity of TREM2 was measured using the ImageJ computer program. (**C**) Protein expression levels of TREM2 according to the pathological T-stage of colon carcinoma tissues.

To explore the role of TREM2 in colon cancer development, we conducted a human colon carcinoma and normal tissue array containing 110 human core colon cancer tissues and 10 normal tissues. The expression levels of TREM2 in the tissue array were visualized by immunohistochemical analysis using a TREM2-specific primary antibody, HRP-conjugated secondary antibody and DAB (3,3′-Diaminobenzidine) and by measuring the intensity using the ImageJ computer program (ver. 1.37). Furthermore, we classified the intensity grades as 1, 2, 3 and 4. We found that normal colon tissues and stage I colon carcinoma contained higher TREM2 protein levels (intensity grades 2 and 3) than stage II, III and IV colon carcinoma (Figure 6A, graph), as shown in the representative images (Figure 6A, right panels). Importantly, TREM2 protein levels decreased gradually in a tumor stage-dependent manner. Furthermore, all 10 of the normal colon tissues contained TREM2 expression at intensity 2, and all 7 of the stage IV colon carcinoma tissues expressed very low levels of TREM2 and were classified as intensity 1 (Figure 6B, graph). Importantly, although TREM2 protein levels were heterogeneous, including intensity grades 1, 2, 3 and 4 for stage II and III (Figure 6B, graph), we found a prominent pattern in which TREM2 protein levels gradually decreased in a tumor stage-dependent manner (stage II: 27 of the 57 samples were classified as intensity 1 (47.4%); stage III: 28 of the 43 samples were classified as intensity 1 (65.1%), and 7 of the 7 samples were classified as intensity 1 (100%) (Figure 6B, graph), as shown in the representative images (Figure 6B, right panels)). According to pathological T-stage classification, we found that although TREM2 protein levels were heterogeneous in T3- and T4-stage samples, TREM2 levels in intensity 1 group were increased to 40%, 49% and 63% in T2-, T3- and T4-stage human colon carcinoma tissues, respectively (Figure 6C). These results indicate that the downregulation of TREM2 may result in greater susceptibility to the development of colon cancer.

## 3. Discussion

Although metastatic colon cancer can respond to chemotherapy and radiotherapy, it is seldom controlled adequately. Therefore, it is important to identify new molecular candidates for colon cancer that may be potential targets for chemotherapy or immunotherapy. TREM2 has been known to regulate the development of osteoclasts [28,29] and is involved in dendritic cell maturation [13]. However, knowledge about the biological function of TREM2 is still limited because specific TREM2 ligands have not been identified. Furthermore, the involvement of TREM2 in the pathophysiology of various cancers, especially colon carcinoma, is completely unknown. In this study, we elucidated a novel molecular mechanism of TREM2-mediated suppression of colon tumorigenicity. We found that TREM2 overexpression suppressed tumor growth in vitro and in vivo via the negative regulation of the Wnt1/*β*-catenin signaling pathway. Importantly, the ability of TREM2 to inhibit the tumorigenicity of colon cancer was further supported by our finding that TREM2 expression in human colon cancer tissues is gradually decreased in a tumor stage-dependent manner. Therefore, we provided the first demonstration that TREM2 is required for the suppression of colon cancer development.

In the present study, TREM2 signaling blockade remarkably increased the S phase but reduced the G0/G1 phase in the cell cycle (Figure 1D). In addition, p21 and p53 cell cycle arrest proteins were downregulated by the blockade of TREM2 (Figure 1E), suggesting that TREM2 suppresses cell cycle progression by upregulating cell cycle arrest proteins. According to previous study, a key point for targeting cells stuck in the S-phase is the specificity for KRas-driven cancer cells, in that these cancer cells do not arrest in the G1 phase [30]. KRas mutations are present in about 30% of all human cancers and KRas-driven cancers have been largely resistant to therapeutic intervention, and KRas itself has been considered undruggable [30]. Therefore, the S-phase checkpoint is an exciting opportunity for therapeutic intervention in KRas-driven cancers because the S phase of the cell cycle is the most carefully modulated phase of the cell cycle, where the cell is replicating its genome and separating the chromosomes [30]. Thus, our findings provide the possibility that the TREM2-mediated suppression of the S phase cell cycle might be useful for therapeutic application in colorectal cancer.

Epithelial cell adhesion molecule (EpCAM), a carcinoma-associated antigen, is known to be overexpressed in a variety of human adenocarcinomas and squamous cell carcinomas, such as colon, stomach, prostate and lung cancers [31]. A previous study demonstrated that Wnt signaling activation induces the upregulation of EpCAM via the GSK3*β*/*β*-catenin pathway [32]. We also found that colon cells from TREM2-TG mice showed decreased protein levels of total EpCAM (Figure S2). Phosphorylation of GSK-3*β* at Ser9 and total *β*-catenin levels (Figure 3B,D) suggested that the suppression of EpCAM might be due to alterations in the TREM2-mediated Wnt/*β*-catenin signaling pathway and that TREM2 might be involved in the tumorigenicity of colon cancer. We therefore investigated the molecular mechanism by which TREM2 regulates the Wnt/*β*-catenin signaling pathway in HT29 colon cancer cells. Previous studies demonstrated that Wnt signaling inhibits the phosphorylation and degradation of *β*-catenin by inducing the phosphorylation and inactivation of GSK-3*β*, resulting in the accumulation of cytoplasmic *β*-catenin and its translocation to the nucleus [18,33]. We found that TREM2 inhibited the canonical Wnt1/*β*-catenin signaling pathway (Figure 3), which in turn suppressed the proliferation and tumorigenicity of HT29 colon cancer cells (Figure 1) by inhibiting *β*-catenin/TCF4 transactivation activity (Figure 3E), resulting in the suppression of cell proliferation and DNA synthesis (Figure 1A–C). These results were well correlated with those in TREM2-TG mice. We also found that TREM2-TG mice showed reduced *β*-catenin protein levels compared with those in WT mice. Furthermore, the gene expression of Wnt and TCF4 was suppressed by the transient expression of TREM2 compared with that in non-transfected or mock-transfected colon cancer cells (Figure 3A). Taken together, these results demonstrated that TREM2 suppressed cell proliferation by attenuating the Wnt/*β*-catenin signaling pathway.

To identify the physiological relationship between TREM2 and human colon cancer, we conducted a human colon cancer tissue array using a TREM2-specific antibody. Surprisingly, our results demonstrated that early-stage colon cancer tissues were characterized by high TREM2 protein levels compared with normal colon tissues (Figure 6). We found that normal colon tissues contained relatively low levels of TREM2 protein. However, TREM2 protein levels were initially increased in stage I (Figure 6A,B). Previous studies demonstrated that tumors exhibit a significant infiltration of immune cells, including myeloid cells expressing TREM2 during tumor initiation and progression [34,35,36]. These reports suggest that TREM2 expression can be increased by myeloid cells infiltrated to tumor tissues in the early stage of colorectal cancer progression. TREM2 protein loss gradually increased dependent on the tumor grade from stage II to stage IV. Specifically, the decrease in TREM2 protein level reached approximately 47% at stage II, approximately 63% at stage III and 100% at stage IV (Figure 6B). Interestingly, although the sample numbers were small, the TREM2 protein level was increased at stage I (Figure 6A,B). Furthermore, stage II and stage III colon cancer tissues harbored TREM2 protein levels that were approximately 25–33% higher than those in normal colon tissues (Figure 6A,B). From these results, we suggest that the loss of TREM2 protein expression might be caused by epigenetic modifications, such as promoter methylation.

Similar to the Wnt pathway, the ERK pathway is also known to participate in the regulation of cell growth and plays an important role in colon cancer tumorigenesis [37,38]. ERK associates with phosphorylate GSK-3*β*, which results in the inactivation of GSK-3*β* and upregulation of *β*-catenin [20]. We also found that the suppression of TREM2 induced Wnt signaling pathway activation (Figure 3B) and that TREM2 inactivated the ERK signaling pathway but not the AKT signaling pathway (Figure 4A). Therefore, the TREM2-mediated Wnt/ERK/GSK-3*β* signaling pathway inhibited the metastasis of HT29 colon cancer cells (Figure 2A,B).

## 4. Materials and Methods

### 4.1. Mice

Female C57BL/6J WT and TREM2-TG mice were used at five to seven weeks of age. All mice were maintained under specific pathogen-free conditions. All animal experiments were carried out in accordance with the guidelines of the Institutional Animal Care Committee of Chonnam National University (CNU IACUC-YB-2017-19).

### 4.2. Tissue Array

A human colon tissue array (Product No BC051110) was purchased from U.S. Biomax Inc. (Rockville, MD, USA), and the analysis was conducted according to the manufacturer’s suggested protocol. The tissue array included 10 normal tissues (J3–J12) and 110 cancer tissues. The slide was baked at 60 °C for 2 h, deparaffinized and rehydrated. Antigens were then retrieved by submerging the slide in boiling sodium citrate buffer (10 mM, pH 6.0) for 10 min. The sample was blocked with 3% BSA in 1X PBS/0.03% Triton X-100 in a humidified chamber for 1 h at room temperature and then incubated with a TREM antibody (reconstituted at 0.2 mg/mL in 1× PBS/0.03% Triton X-100) at 4 °C in a humidified chamber overnight. The slide was washed, hybridized with a secondary antibody (anti-goat, chicken antibody) conjugated with horseradish peroxidase (HRP, 1:1000) for 1.5 h at room temperature in the dark. The protein levels were visualized by colorimetric development of HRP using ImmPACT™ DAB with a metal enhancer (Vector Labs #SK-4105). The tissues were observed under a light microscope (X200).

### 4.3. Generation of TREM2-TG Mice

A forward primer (5′-GAA TTC GCC CTT GGC TGG CTG CTG GCA-3′) and reverse primer for the mouse TREM2 nucleotide sequence (5′-GTA CGT GAG AGA ATT C-3′) were used to amplify TREM2 cDNA by RT-PCR. The amplified cDNA was cloned into the pGEM (Professional Group on Engineering Management)-T vector (Promega, Madison, WI), digested with the EcoRI restriction enzyme and then cloned into the pcDNA3.1 expression vector (Invitrogen, Carlsbad, CA, USA) to construct the recombinant expression vector pcDNA3.1/TREM2. Pure isolated recombinant TREM-2 cDNA was microinjected into fertilized eggs (0.5 days) from C57BL/6J mice, and the eggs were then implanted into the oviducts of pseudopregnant C57BL/6J female mice that had been mated with vasectomized male mice of the same strain 1 day before. TREM2-TG mice highly expressed TREM2 in the various tissues, such as the liver, lung, brain, heart, thymus, spleen, colon and bone marrow compared with WT mice [39]. However, no specific difference in phenotypes and behaviors between WT and TREM2-TG mice was observed [40].

### 4.4. Cell Lines, Reagents and Antibodies

HT29 and MC38 colon cancer cell lines were grown in McCoy’s medium and DMEM containing 10% heat-inactivated fetal bovine serum (FBS) at 37 °C in a humidified 5% CO_2_ incubator. Cell viability was measured using a 3-(4,5-dimethylthiazol-2-yl)-5-(3-carboxymethoxyphenyl)-2-(4-sulphophenyl)-2H-tetrazolium) (MTS) assay kit (Promega), as described in the manufacturer’s protocol. Lithium chloride (LiCl), PD98509 and okadaic acid were purchased from Sigma (St. Louis, MO, USA). Antibodies for Western blot analysis were obtained from Cell Signaling Technology: anti-GSK-3*β*, anti-phosphorylated GSK-3β (anti-p-GSK-3*β*), anti-AKT, anti-phosphorylated AKT (anti-p-AKT), anti-*β*-catenin, anti-phosphorylated *β*-catenin (anti-p-*β*-catenin), anti-phosphorylated ERK (anti-p-ERK) and anti-ERK. Antibodies against Wnt, p53 and p21 were obtained from Santa Cruz Biotechnology (Santa Cruz, CA, USA). Anti-TREM2 and TREM2-Ig were obtained from R&D Systems (Abingdon, UK). Anti-EpCAM and anti-*a*-tubulin antibodies were purchased from Abcam (Cambridge, UK) and Sigma, respectively. Horseradish peroxidase (HRP)-conjugated anti-goat IgG and anti-rabbit IgG (Santa Cruz Biotechnology, Dallas, TX, USA) were used as secondary antibodies for Western blot analysis.

### 4.5. Isolation of Primary Colon Cells

Whole colon tissue was removed and dissected away from any attached connective tissues. The tissues were cut longitudinally into 0.5~1-cm lengths in 10 cm^2^ culture dishes containing ice-cold RPMI (Roswell Park Memorial Institute)-1640 and gently washed twice with wash solution (RPMI-1640 supplemented with 2% FBS). The samples were transferred to a 50 mL conical tube containing 20 mL of RPMI-1640 plus type IV collagenase (1 mg/mL) and incubated in a 37 °C shaking incubator at 250 rpm for 15 min. They were passed through a 100-mesh sieve and centrifuged at 1700 rpm for 5 min at room temperature. The remaining tissues were then re-incubated in 20 mL of RPMI plus collagenase (1 mg/mL) in a 37 °C shaking incubator at 250 rpm for 15 min. These steps were repeated five more times, and the pellet was resuspended in RPMI medium supplemented with 10% FBS. The isolated primary colon cells were used for western blot analysis and RT-PCR.

### 4.6. Cell Transfection

HT29 cells were plated 24 h before transfection at 60% confluency and transiently transfected with the pcDNA3.1(+) vector alone or the same vector containing TREM2 cDNA using Lipofectamine 2000 (Invitrogen, Carlsbad, CA, USA) as described in the manufacturer’s protocol. At 4 h post-transfection, the DNA-containing medium was replaced with fresh medium supplemented with 10% FBS, and the cells were incubated at 37 °C in a humidified 5% CO_2_ atmosphere for 24 h, followed by cell harvesting for various analyses.

### 4.7. Reverse Transcription-polymerase Chain Reaction (RT-PCR)

Total cellular RNA was extracted using TRIZOL^®^ reagent (MRC, Cincinnati) according to the manufacturer’s instructions. Aliquots (5 μg) of the RNA were transcribed into cDNA at 42 °C for 1 h in a total volume of 50 μL with 2.5 U of Moloney murine leukemia virus (MMLV) reverse transcriptase (RT), 10 mM of each dNTP (Deoxyribonucleotides) and 10 pmol oligo (dT) primers in 5× RT buffer (Promega). Reverse-transcribed cDNA samples were added to a PCR mixture containing 10× PCR buffer, 0.25 mM dNTP, 0.5 U of Taq DNA polymerase and 10 pmol of primers for each gene. The primer sequences were as follows: m.TREM2, 5′-ATG GGA CCT CTC CAC CAG TT-3′ and 5′-GGG TCC AGT GAG GAT CTG AA-3′; h.TREM2, 5′-TGC CCA TGT GGA GCA CAG CAT CTC-3′ and 5′-CTC CCC ACT CCC TCA ACC AGT CCC-3′; h.DAP12, 5′-GGG ATC CCG GCT GGT CCC T-3′ and 5′-CGA ATT CTG TCA TGA TTC GGG C-3′; m.DAP12, 5′-ATG GGG GCT CTG GAC CCC T-3′ and 5′-TCA TCT GTA ATA TTG CCT CT-3′; h,m.β-actin, 5′-GTG GGG CGC CCC AGG CAC CA-3′ and 5′-CTC CTT AAT GTC ACG CAC GAT-3′; m.MMP9, 5′-CGT GTC TGG AGA TTC GAC TTG A-3′ and 5′-TGG AAG ATG TCG TGT GAG-3′; h.MMP9, 5′-TGT ACC CTA TGT ACC GCT TC-3′ and 5′-TCC CAT CCT TGA ACA AAT AC; h.TCF4, 5′-TCA CCA ACA GCG AAT GGC-3′ and 5′-AGG AAG GAT AGC CTG GCG-3′; h.Wnt1, 5′-GCT TCC TCA TGA ACC TTC ACA-3′ and 5′-GCG ATT TCT CGA AGT AGA CCA G-3′. The primer sequences for Figure S1B were described in Table S1. All PCR mixtures were denatured at 95 °C for 1 min and cycled 27 times for *β*-actin and 30 times for all other reactions at 95 °C for 1 min, 56 °C for 1 min, and 72 °C for 2 min, followed by an additional extension step at 72 °C for 10 min. The PCR products were electrophoresed and visualized by ethidium bromide staining.

### 4.8. Western Blot Analysis

Cells were lysed in protein lysis buffer (20 mM HEPES (N-2-hydroxyethylpiperazine-N-ethanesulfonic acid) (pH 7.9), 100 mM KCl, 300 mM NaCl, 10 mM EDTA, 0.5% Nonidet P-40, 1 mM Na3VO4, 1 mM PMSF, 10 µg/mL aprotinin, and 10 µg/mL leupeptin) for 30 min on ice. The protein concentrations were measured using Bradford reagent (Bio-Rad). Cell lysates containing equal amounts of protein were subjected to sodium dodecyl sulfate-polyacrylamide gel electrophoresis (SDS-PAGE) and transferred to an immunoblot polyvinylidene fluoride (PVDF) membrane (Millipore, Marlborough, MA). The membrane was incubated with blocking buffer (1% BSA and 5% skim milk in PBS) at 4 °C overnight and then washed three times with TBS-T (Tris buffered saline-tween 20) (50 mM Tris (pH 7.4), 150 mM NaCl, 0.05% Tween 20). The membrane was incubated with the individual antibodies at room temperature for 2 h, followed by incubation with HRP-conjugated secondary antibodies at room temperature for 1 h. After washing three times with TBS-T, the immunoblotted proteins were detected using an ECL (enhanced chemiluminescence) system (Millipore).

### 4.9. Cell Proliferation and Colony Forming Assays

HT29 cells (2 × 10^5^) were cultured in a 96-well plate and treated with human-Ig (H-Ig) or TREM2-Ig (T-Ig) for 2 days, and proliferation was determined using a CellTiter 96^®^ AQueous One Solution Cell Proliferation Assay (Promega) as described in the manufacturer’s protocol. For the colony forming assay, HT29 cells (1 × 10^4^) were cultured in a six-well plate. After treatment with H-Ig or T-Ig for 10 days, the cells were fixed with 4% paraformaldehyde for 20 min. The cells were then washed with PBS and stained with a 0.5% Coomassie blue solution.

### 4.10. Cell Cycle Analysis

HT29 cells (2 × 10^5^ cells/6-cm^2^ culture dish) were fixed in cold 75% ethanol at 4 °C overnight and washed twice with PBS. The cells were then stained with 0.5 μL of 10 mg/mL propidium iodide (PI) containing 0.1 mg/mL RNase A for 30 min at room temperature. DNA content in the cells was analyzed by flow cytometry (BD Biosciences, San Diego, CA, USA) using Modfit LT 3.0 software (Verity Software House, San Diego, CA, USA).

### 4.11. Luciferase Reporter Assays

HT29 cells (5 × 10^4^ cells) were cotransfected with 1 μg of either TOPflash or FOPflash luciferase reporter plasmids (Upstate Biotechnology, Lake Placid, NY, USA) using Lipofectamine 2000. For each transfection, 0.4 μg of *β*-galactosidase cDNA was cotransfected to normalize the transfection efficiency. After 24 h, the cells were lysed with luciferase lysis buffer [1% Triton X-100, 2 mM EDTA (pH 8.0), 15 mM MgSO_4_, 2.5 mM glycine] for 20 min. The luciferase activity was measured using luciferin (Promega) and analyzed using a MicroLumat Plus LB96V luminometer (Berthold Technologies, Bad Wildbad, Germany).

### 4.12. Scratch Wound-healing Assay

To investigate the migration ability of HT29 cells, HT29 cells were seeded on six-well culture plates at a density of 1 × 10^5^ cells per well and allowed to grow to confluence over 24 h. The confluent monolayers were scratched with a pipette tip and cultured for 5 days. The plates were washed once with medium to remove the nonadherent cells and photographed under a phase-contrast microscope.

### 4.13. Matrigel Invasion Assay

Matrigel invasion assays were performed with BioCoat™ Matrigel Invasion Chambers (BD Biosciences, Franklin Lakes, NJ, USA) according to the manufacturer’s protocol. HT29 cells (3 × 10^5^ cells) were resuspended in 100 µL of serum-free medium and added to the upper compartment of the transwell invasion chamber (8 µm pores). Next, 800 µL of fresh complete medium was added to the lower compartment of the chamber as a chemoattractant and incubated for 24 h. After wiping the cells off the upper compartment of the chamber with a cotton swab, the cells that invaded to the lower surface of the filter were fixed with 100% methanol. The invasive cells were then stained with hematoxylin and eosin (Sigma) and counted in five randomly selected microscopic fields (100×) per filter.

### 4.14. MC38 Cancer Growth in Mice

WT littermate or TREM2-TG mice were injected subcutaneously with 5 × 10^5^ MC38 cells. During the observation period, the tumor volumes were measured by bilateral Vernier calipers and calculated using the following formula: width × length × height × π/6.

### 4.15. Statistical Analysis

For the statistical analysis of the data, *P* values were determined using a two-tailed Student’s *t* test software program (Startview 5.1; Abacus Concepts, Berkeley, CA, USA). The results were considered statistically significant when *p* values were <0.05.

## 5. Conclusions

In conclusion, our results strongly suggest that TREM2 is highly involved in the malignancy of colon cancer cells but not in the cell transformation stage. Therefore, TREM2 may act as an indicator and/or potential new therapeutic target for colon cancer.

## Supplementary Materials

The following are available online at https://www.mdpi.com/2072-6694/11/9/1315/s1, Figure S1: Infiltrated immune cell distribution and cytokine mRNA expression in tumors, Figure S2: Reduced protein expression of EpCAM in TREM2-TG mice compared with that in WT mice, Table S1: Real-time-PCR primer sequences.
