# Supplementary material for: TREM2 Acts as a Tumor Suppressor in Colorectal Carcinoma through Wnt1/β-catenin and Erk Signaling
Source: Cancers (Basel). 2019 Sep 6;11(9):1315. doi: 10.3390/cancers11091315 (PMC6770495; doi:10.3390/cancers11091315)
Supplement: Supplementary file 1 [file cancers-11-01315-s001.pdf]

Supplementary Materials

Table S1. Real-time-PCR primer sequences.

Gene	Forward	Reverse
IL-1β	5'-CAACCAACAAGTGATATTCTCCATG-3'	5'-GATCCACACTCTCCAGCTGCA -3'
IL-4	5'-ACAGGAGAAGGGACGCCAT-3'	5'-GAAGCCCTACAGACGAGCTCA-3'
IL-6	5'-CCGGAGAGGAGACTTCACAG-3'	5'-CAGAATTGCCATTGCACAAC-3'
IL-12	5'-ATGACCCTGTGCCTTGGTAG-3'	5'-CAGATAGCCCATCACCCTGT-3'
IL-15	5'-CATCCATCTCGTGCTACTTGTGTT-3'	5'-CATCTATCCAGTTGGCCTCTGTTT-3'
IL-17a	5'-TCAGACTACCTCAACCGTTCC-3'	5'-CTTTCCCTCCGCATTGACAC-3'
IL-21	5'-GCCAAACTCAAGCCATCAAACCC-3'	5'-CGAATCACAGGAAGGGCATTTAGC-3'
INF-γ	5'-TGGCATAGATGTGGAAGAAAAGAG-3'	5'-TGCAGGATTTTCATGTCACCAT-3'

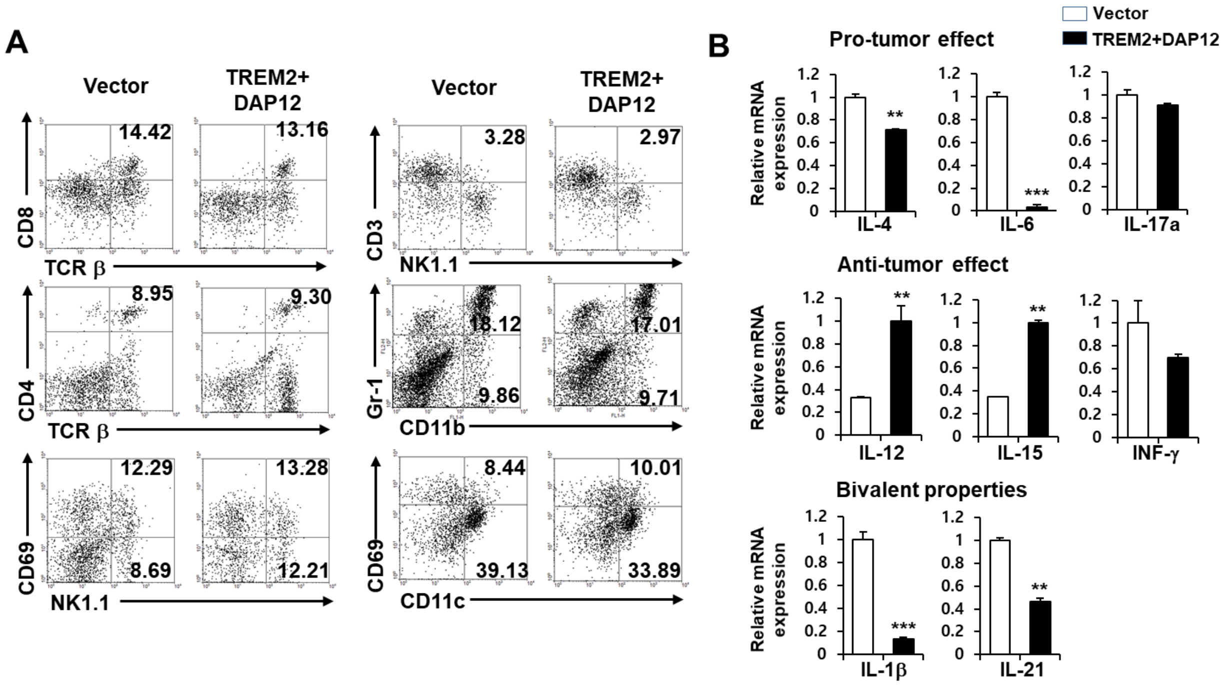

Figure S1. Infiltrated immune cell distribution and cytokine mRNA expression in tumors. **(A)** Cells were isolated from tumors, and the immune cell population was determined by flow cytometry analysis using the indicated antibodies (CD4 T cells: $CD4^{+}TCR\beta^{+}$; CD8 T cells: $CD8^{+}TCR\beta^{+}$; NK cells: $NK1.1^{+}$; NK T cells: $NK1.1^{+}CD3^{+}$; macrophages: $CD11b^{+}$; granulocytes: $CD11b^{+}Gr\text{-}1^{+}$; dendritic cells: $CD11c^{+}$; activation marker: $CD69^{+}$). **(B)** cDNA was synthesized from tumor mRNA and analyzed by real-time PCR using the primers indicated in Table 1. The data are representative of three independent experiments (**p<0.01, ***p<0.001).

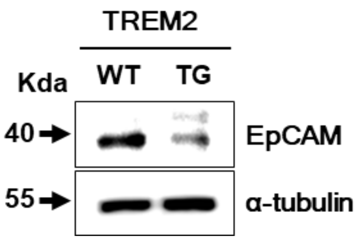

Figure S2. Reduced protein expression of EpCAM in TREM2-TG mice compared with that in WT mice. EpCAM expression was measured by western blot analysis using protein from the colon tissue of TREM2-TG and WT mice.
